# Limited population structure but signals of recent selection in introduced African Fig Fly (*Zaprionus indianus*) in North America

**DOI:** 10.1093/g3journal/jkaf178

**Published:** 2025-08-07

**Authors:** Priscilla A Erickson, Alexandra Stellwagen, Alyssa Bangerter, Ansleigh Gunter, Nikolaos T Polizos, Alan O Bergland

**Affiliations:** Department of Biology, University of Richmond, Richmond, VA 23173, United States; Department of Biology, University of Richmond, Richmond, VA 23173, United States; Department of Biology, University of Virginia, Charlottesville, VA 22903, United States; Department of Biology, University of Richmond, Richmond, VA 23173, United States; Department of Biology, University of Miami, Coral Gables, FL 33146, United States; Department of Biology, University of Virginia, Charlottesville, VA 22903, United States

**Keywords:** *Zaprionus indianus*, invasive species, population genomics, population structure, selective sweep

## Abstract

Invasive species have devastating consequences for human health, food security, and the environment. Many invasive species adapt to new ecological niches following invasion, but little is known about the early steps of adaptation. Here, we examine the population genomics of a recently introduced drosophilid in North America, the African Fig Fly, *Zaprionus indianus*. This species is likely intolerant of subfreezing temperatures and recolonizes temperate environments yearly. We generated a new chromosome-level genome assembly for *Z. indianus*. Using resequencing data of over 200 North American individuals collected over 4 years in temperate Virginia, along with a single collection from subtropical Florida, we tested for signatures of population structure and adaptation within invasive populations. We show that founding populations are sometimes small and contain close genetic relatives, yet temporal population structure and differentiation of populations are mostly absent across North America. However, we identify 2 haplotypes that are differentiated between African and invasive populations and show signatures of selective sweeps. Both haplotypes contain genes in the cytochrome P450 pathway, indicating these sweeps may be related to pesticide resistance. X chromosome evolution in invasive populations is strikingly different from the autosomes, and a haplotype on the X chromosome that is differentiated between Virginia and Florida populations is a candidate for temperate adaptation. These results show that despite limited population structure, populations may rapidly evolve genetic differences early in an invasion. Further uncovering how these genomic regions influence invasive potential and success in new environments will enhance our understanding of how organisms evolve in changing environments.

## Introduction

Understanding how species expand and adapt to new environments in an era of changing land use, environmental changes, and global commerce is central to controlling the spread of disease (Altizer et al. 2013; Hoberg and Brooks 2015), maintaining crop security (Oerke 2006; Sutherst et al. 2011), and preserving biodiversity (Bellard et al. 2012). Many organisms are moving to new, previously unoccupied ranges at rates that continue to accelerate (Ricciardi 2007; Seebens et al. 2015, 2017; Platts et al. 2019; Sardain et al. 2019) due to changing environmental conditions, habitat alteration, and anthropogenic introductions. Genetic adaptation to new environments may allow some vulnerable organisms to survive in new habitats but may also permit potentially harmful organisms to expand even further (Clements and Ditommaso 2011). The past 2 decades have produced a wealth of studies characterizing the genetic and genomic basis of adaptation in a variety of organisms, from experimental populations of microbes (Good et al. 2017; Nguyen Ba et al. 2019; Johnson et al. 2021) to natural populations of eukaryotes (Hancock et al. 2011; Jones et al. 2018; Barrett et al. 2019; Lovell et al. 2021; Schluter et al. 2021). Recent and ongoing invasions offer the opportunity to study rapid evolution and adaptation to new environments in nearly real-time (Koch et al. 2020; Pélissié et al. 2022; Parvizi et al. 2023; Soudi et al. 2023). Recently, genomics has helped trace the history and sources of many well-known invasions (Pélissié et al. 2022; Picq et al. 2023) and shown that genetic divergence and even local adaptation are common in invasive populations that have been established for decades or even centuries (Ma et al. 2020; Stuart et al. 2021; Li et al. 2023). However, much remains unknown about the role that evolution plays in allowing invasive organisms to colonize and thrive in new environments. A better understanding of adaptive pathways in invasion may assist in predicting the success of invasions and controlling their outcomes.

The African Fig Fly, *Zaprionus indianus*, serves as a unique model to study how invasion history and local environment influence patterns of genetic variation. The ongoing, recurrent invasion of *Z. indianus* in North America offers a premier opportunity to study the possibility of rapid genetic changes following invasion. The *Zaprionus* genus arose in Africa, but *Z. indianus* was first described in India in 1970 (Gupta 1970), where it has adapted to a range of environments (da Mata et al. 2010). It is one of the most ecologically diverse drosophilids in Africa; its ability to utilize up to 80 different food sources (Yassin and David 2010) and its generation time of as few as ∼13 d (Nava et al. 2007) likely fueled its spread around the world. In 1999, it was first detected in Brazil (Vilela 1999), where it subsequently spread and caused major damage to fig and berry crops as well as native fruit species (Leão and Tldon 2004; Oliveira et al. 2013; Roque et al. 2017; Zanuncio-Junior et al. 2018; Allori Stazzonelli et al. 2023). It was later found in Mexico and Central America between 2002 and 2003 (Markow et al. 2014) and eventually Florida, United States, in 2005 (van der Linde et al. 2006). Between 2011 and 2012, its range expanded northwards in eastern North America (Joshi et al. 2014; Van Timmeren and Isaacs 2014; Pfeiffer et al. 2019) and eventually reached as far north as Ontario, Canada (Renkema et al. 2013) and Minnesota, United States (Holle et al. 2019). It has also recently been found in the Middle East, Europe, and Hawaii (Parchami-Araghi et al. 2015; Kremmer et al. 2017; Willbrand et al. 2018), suggesting that the invasion is ongoing. *Z. indianus* can damage soft-skinned fruit crops (Bernardi et al. 2017; Pfeiffer et al. 2019; Allori Stazzonelli et al. 2023), increasing concerns about its pest potential in its expanding range.

Despite its global success, *Z. indianus* males are sterile below 15 °C, making cold temperatures a limiting factor to their success (Araripe et al. 2004). Within the temperate environment of Virginia, the species exhibits strong seasonal fluctuations in abundance (Rakes et al. 2023). First detection in Virginia is usually in July, weeks after the appearance of other overwintering drosophilids, and population sizes climb dramatically through the late summer and early fall, when it often dominates the drosophilid community in temperate orchards. Typically, peak abundance of *Z. indianus* occurs in early to mid-September and is followed by a dip in abundance and then a second peak in October, suggesting a seasonal component to reproduction or fluctuations in factors influencing its relative fitness. However, despite its early post-colonization success, it does not appear to survive temperate winters; *Z. indianus* populations became undetectable in Virginia by early December (Rakes et al. 2023). *Z. indianus* were not collected during regular winter sampling in Memphis, Tennessee (Kohlmeier and Kohlmeier 2025), and they were collected in lower numbers during the winter in northern Florida relative to central Florida (Renkema et al. 2018), suggesting that even mild winters (only 8 nights below 0 °C) can substantially reduce their numbers. *Z. indianus* has been detected in locations in Minnesota, Kansas, and the northeastern United States in one year but not the next, suggesting that the populations may be extirpated by cold and re-introduced by stochastic dispersal processes (Gleason et al. 2019; Holle et al. 2019; Rakes et al. 2023). While conclusive data are still lacking, these results collectively suggest *Z. indianus* likely repeatedly invades temperate environments each year from warmer refugia. Reinvasion offers an opportunity to recurrently study the genetic impacts of invasion and the potential for post-colonization adaptation across multiple years of sampling.

Genetic studies *of Z. indianus* are limited but provide important context to understand its worldwide invasion. The invasion of North America likely resulted from separate founding events on the East and West coasts (Commar et al. 2012). Comeault et al. (2020) showed that North American populations are genetically distinct from those from Africa. Invasive populations of *Z. indianus* have an approximately 30% reduction in genetic diversity relative to ancestral African populations (Comeault et al. 2020), though invasive populations of *Z. indianus* maintain levels of genetic diversity that are often higher than those of non-invasive congeners. Despite the loss of diversity, *Z. indianus* is extremely successful in temperate habitats (Rakes et al. 2023) and has competitive advantages over other drosophilids (Walsh-Antzak and Erickson 2025). Further studies demonstrated that genetically distinct populations from eastern and western Africa likely admixed prior to a single colonization of the Americas (Comeault et al. 2021). How the high degree of genetic diversity in invasive populations influences the potential for ongoing evolution in North America, which is in a critical early stage of invasion, remains understudied.

Here, we assembled and annotated a chromosome-level genome assembly for *Z. indianus* and used the newly improved genome to answer several questions with the whole genome sequences of over 200 North American flies collected from 3 locations over 4 years. First, do recolonizing North American *Z. indianus* populations demonstrate spatial or temporal population structure, and if so, do specific regions of the genome have an outsized contribution to population structure? Second, is the invasion and recolonization history recapitulated in population genetic data? And third, do temperate populations show signatures of selection relative to native and tropical invasive populations?

## Materials and methods

### Hi-C based genome scaffolding

An inbred line was generated from flies originally captured from Carter Mountain Orchard, Virginia (37.9913°N, 78.4721°W) in 2019. Wild caught flies were reared in the lab for approximately 1 year prior to initiating isofemale lines. The offspring of the isofemale lines were propagated through 10 rounds of full-sib mating. The resulting lines were then passaged for approximately one additional year in the lab and the most vigorous remaining line (“24.2”) was chosen for sequencing.

Third instar larvae from a single inbred line were snap frozen in liquid nitrogen and sent to Dovetail corporation (now Cantata Bio, Scotts Valley, CA) for chromatin extraction, Hi-C sequencing, and genome scaffolding. Briefly, chromatin was fixed in place with formaldehyde in the nucleus and then extracted. Fixed chromatin was digested with DNase I, chromatin ends were repaired and ligated to a biotinylated bridge adapter followed by proximity ligation of adapter-containing ends. After proximity ligation, crosslinks were reversed, and the DNA was purified. The purified DNA was treated to remove biotin that was not internal to ligated fragments. Sequencing libraries were generated using NEBNext Ultra enzymes and Illumina-compatible adapters. Biotin-containing fragments were isolated using streptavidin beads before PCR enrichment of each library. The library was sequenced on an Illumina HiSeqX platform to produce approximately 30 × sequence coverage.

The input de novo assembly was the *Z. indianus* “RCR04” PacBio assembly (assembly # ASM1890459v1) from Kim et al. (2021). This assembly and Dovetail OmniC library reads were used as input data for *HiRise*, a software pipeline designed specifically for using proximity ligation data to scaffold genome assemblies (Putnam et al. 2016). Dovetail OmniC library sequences were aligned to the draft input assembly using *bwa* (Li and Durbin 2009). The separations of Dovetail OmniC read pairs mapped within draft scaffolds were analyzed by *HiRise* to produce a likelihood model for genomic distance between read pairs, and the model was used to identify and break putative misjoins, score prospective joins, and make joins above a threshold. See Supplementary Fig. 1 for link density histogram of scaffolding data.

### Annotation

Repeat families found in the genome assemblies of *Z. indianus* were identified de novo and classified using the software package *RepeatModeler* v. 2.0.1 (Flynn et al. 2020). *RepeatModeler* depends on the programs *RECON* v. 1.08 (Bao and Eddy 2002) and *RepeatScout* v. 1.0.6 (Price et al. 2005) for the de novo identification of repeats within the genome. The custom repeat library obtained from *RepeatModeler* was used to discover, identify, and mask the repeats in the assembly file using *RepeatMasker* v. 4.1.0 (Smit et al. 2015).

RNA sequencing was conducted on 3 replicates of third instar larva and 3 replicates of mixed stage pupa that were snap frozen in liquid nitrogen. RNA extraction and sequencing was performed by GeneWiz (South Plainfield, NJ). New larval and pupal RNAseq reads were combined with adult RNA sequencing from Comeault et al. (2020) for annotation. Coding sequences from *D. grimshawi*, *D. melanogaster*, *D. pseudoobscura*, *D. virilis*, *Z. africanus*, *Z. indianus*, *Z. tsacasi*, and *Z. tuberculatus* (Kim et al. 2021) were used to train the initial *ab initio* model for *Z. indianus* using the *AUGUSTUS* software v. 2.5.5 (Keller et al. 2011). Six rounds of prediction optimization were done with the software package provided by *AUGUSTUS*. The same coding sequences were also used to train a separate *ab initio* model for *Z. indianus* using *SNAP* (version 2006-07-28) (Korf 2004). RNAseq reads were mapped onto the genome using the *STAR* aligner software (version 2.7) (Dobin et al. 2013) and intron hints generated with the *bam2hints* tools within *AUGUSTUS*. *MAKER* v. 3.01.03 (Cantarel et al. 2008), *SNAP*, and *AUGUSTUS* (with intron–exon boundary hints provided from RNAseq) were then used to predict for genes in the repeat-masked reference genome. To help guide the prediction process, Swiss-Prot peptide sequences from the UniProt database were downloaded and used in conjunction with the protein sequences from *D. grimshawi, D. melanogaster, D. pseudoobscura, D. virilis, Z. africanus, Z. indianus, Z. tsacasi, and Z. tuberculatus* to generate peptide evidence in the *MAKER* pipeline. Only genes that were predicted by both *SNAP* and *AUGUSTUS* were retained in the final gene sets. To help assess the quality of the gene prediction, AED scores were generated for each of the predicted genes as part of the *MAKER* pipeline. Genes were further characterized for their putative function by performing a BLAST search of the peptide sequences against the UniProt database. tRNAs were predicted using the software *tRNAscan-SE* v. 2.05 (Chan and Lowe 2019). Transcriptome completeness was assessed with *BUSCO* v. 4.0.5 (Manni et al. 2021) using the eukaryota_odb10 list of 255 genes.

### Wild fly collections

Flies were collected by aspiration and netting from Carter Mountain Orchard, Virginia from 2017 to 2020 and from Hanover Peach Orchard, Virginia, from 2019 to 2020. Flies were sampled from Coral Gables, Florida, in June 2019 using traps baited with bananas, oranges, yeast, and red wine. See Table 1 for the number of individual flies sequenced from each location and timepoint. Flies were frozen in 70% ethanol at −20 °C (2017 to 2018) or dry at −80 °C (2019 to 2020) prior to sequencing. Collections performed in July and August were called “early season.” In 2019, the earliest collections were not made until September (typically when *Z. indianus* abundance peaks, Rakes et al. 2023) and were assigned “mid-season.” Collections from October and November were called “late season.” For some analyses, the mid-season collection and early collections were combined, as they were the first collections available each year.

**Table 1.** Sample information for new samples collected for this study.

Location	Latitude, longitude	Year	Season	Sampling date (# flies)	Number sequenced	Number after filtering
Coral Gables, FL, United States (FL)	25.535, −80.493	2019	—	1 Jun	25	24
Carter Mountain, Virginia, United States (VA-CM)	37.991, −78.472	2017	early	6 Jun (1) 22 Jun (7) 7 Jul (12)	20	17
		2017	late	9 Nov	20	19
		2018	early	5 Jul (2) 12 Jul (1) 19 Jul (1) 26 Jul (3) 2 Aug (1) 16 Aug (12)	20	20
		2018	late	1 Nov	20	19
		2019	mid	6 Sep	20	20
		2019	late	26 Oct	20	14
		2020	early	17 Jul	20	18
Hanover Peach Orchard, Virginia, United States (VA-HPO)	37.572, −77.266	2019	mid	3 Sep	20	19
		2020	early	16 Jul (5) 3 Aug (25)	30	29

See Supplementary Table 1 for previously sequenced samples incorporated into this analysis.

### Individual whole genome sequencing

The sex of each wild-caught fly was recorded, then DNA was extracted from individual flies using the DNAdvance kit (Beckman Coulter, Indianapolis, IN) in 96-well plates, including an additional RNase treatment step. DNA concentration was measured using the QuantIT kit (Invitrogen, Waltham, MA), and the purified DNA was diluted to 1 ng/µL. Libraries were prepared from 1 ng of genomic DNA using a reduced-volume dual-barcoding Nextera (Illumina, San Diego, CA) protocol, as previously described (Erickson et al. 2020). The libraries were quantified using the QuantIT kit, and equimolar ratios of each individual DNA were combined for sequencing. The pooled library was size-selected for 500 bp fragments using a BluePippin gel cassette (Sage Sciences, Beverly, MA). The pooled libraries were sequenced in one Illumina NovaSeq 6000 lane using paired-end, 150 bp reads by Novogene (Sacramento, CA).

Existing raw reads from *Z. indianus* collections from North America, South America, and Africa (Supplementary Table 1; Comeault et al. 2020, 2021) were downloaded from the sequence read archive (SRA) under BioProject number PRJNA604690. These samples were combined with the new sequence data and processed together with the same mapping and SNP-calling pipeline. Overlapping paired-end reads were merged with *BBMerge* v. 38.92 (Bushnell et al. 2017). Reads were mapped to the genome assembly described above using *bwa mem* v. 0.7.17 (Li and Durbin 2009 ). Bam files for merged and unmerged reads were combined, sorted, and de-duplicated with *Picard* v. 2.26.2 (https://github.com/broadinstitute/picard).

We next used *Haplotype Caller* from *GATK* v. 4.2.0.0 (McKenna et al. 2010) to generate a gVCF for each individual. We built a GenomicsDBI database for each scaffold, then used this database to genotype each gVCF. We used *GATK's* hard filtering options to filter the raw SNPs based on previously published parameters (-*filter-expression* “*QD < 2.0 || FS > 60.0 || SOR > 3.0 || MQ < 40.0 || MQRankSum <* −*12.5 || ReadPosRankSum <* −*8.0*”) (Comeault et al. 2020). We then removed SNPs within 20 bp of an indel from the output and removed all SNPs in regions identified by *RepeatMasker*. We analyzed several measures of individual and SNP quality using *VCFtools* v. 0.1.17 (Danecek et al. 2011). We removed 16 individuals with mean coverage <7 × or over 10% missing genotypes. Next, we filtered SNPs with mean depth <10 or > 50 across all samples. We removed individual genotypes supported by 6 or fewer reads or with more than 100 reads to produce a final VCF with 5,185,389 SNPs and 2,099,147 non-singleton SNPs. See Table 1 and Supplementary Fig. 1 for the final number of individuals included in the analysis from each population. See Supplementary Fig. 2 for the average SNP depth per sampling time and location.

### Sex chromosome and Muller element identification

*samtools* v. 1.12 (Li et al. 2009) was used to measure coverage and depth of mapped reads from individual sequencing. This analysis revealed that the 5 main scaffolds (all over 25 Mb in length) had a mean depth of ∼16 × coverage in both males and females in our dataset, except for scaffold 3, which had ∼16 × coverage in females but ∼8 × coverage in males, suggesting that it is the X chromosome (Supplementary Fig. 3). Some of the previously sequenced samples had no sex recorded, so we used the ratio of X chromosome reads (scaffold 3) to autosome (scaffolds 1, 2, 4, and 5) reads to assign sexes to those individuals. Individuals with a ratio >0.8 were assigned female, and ratios <0.8 were assigned male (Supplementary Fig. 4). For 2 known-sex individuals, the sex recorded prior to sequencing did not match the sex based on coverage; for those 2 samples, we used the coverage-based sex assignment for analyses. We used *D-GENIES* (Cabanettes and Klopp 2018) to create dot-plots comparing the *Z. indianus* and *D. melanogaster* genomes (BDGP6.46, downloaded from ensemble.org) to confirm the sex chromosome identification and assign Muller elements to *Z. indianus* autosomes (Supplementary Fig. 5, Supplementary Table 2). Five additional scaffolds had lengths over 1 Mb. Scaffold 8 is the dot chromosome (Muller element F) based on sequence comparison to *D. melanogaster* (Supplementary Fig. 5) and had similar coverage to the autosomes (Supplementary Fig. 3). Scaffolds 6, 7, 9, and 10 had reduced coverage (Supplementary Fig. 3) and contain mostly repetitive elements. Downstream SNP calling and population genetic analysis included the 5 large scaffolds (named chromosomes 1 to 5 from largest to smallest) and excluded all smaller scaffolds.

### Testing for structural variants

We used *smoove* v. 0.2.6 (Pedersen et al. 2020) to identify and genotype insertions, deletions, and rearrangements in the paired-end sequencing data from all individuals, as described in the documentation. As an alternative approach to search for large structural variants, we used linkage disequilibrium (LD) of randomly sampled SNPs from each chromosome to visually inspect for linkage due to potential inversions. We generated a list of SNPs segregating in each focal population with no missing genotypes and randomly sampled 4,000 SNPs from each chromosome. We used the *snpgdsLDMat* function in the R package *SNPRelate* v. 1.38.0 (Zheng et al. 2012) in R v. 4.1.1 (R Core Team) to calculate LD between all pairs of SNPs. LD heatmaps were created with the *ggLD* package (https://github.com/mmkim1210/ggLD).

To define approximate inversion regions for SNP filtering, we looked for regions with evidence of high long-distance LD within North American samples. We note that our purpose here was to roughly define inversion regions to exclude from population structure analyses, not to precisely define inversion breakpoints, which will likely require further long-read sequencing. For each SNP segregating in North America with complete genotyping information, we randomly chose a second SNP that was 100, 200, 300, 400, or 500 kb away from the focal SNP (±5%). We calculated LD between the pair of SNPs. We then divided the genome in 100 kb nonoverlapping windows and for each window determined if at least 1 SNP in that window showed evidence of high long-distance LD (*R*^2^ > 0.75) at any of the sampled distances. Because some windows contain spurious examples of long-distance LD, we defined inversion regions by looking for consecutive strings of at least 10 windows (1 Mb total) containing high LD SNPs. This approach identified large potential inversion regions on chromosomes 1, 2, 4, and 5 that corresponded to regions visually identified in LD heatmaps. We masked SNPs in these regions (82.6 Mb total) for some downstream analyses.

### Population structure

We conducted principal components analysis (PCA) using *SNPRelate* with a vcf that excluded singleton SNPs. We LD pruned SNPs with minor allele counts of at least 3 (Linck and Battey 2019) using *SNPgdsLDpruning* with an LD threshold of 0.2 and then calculated principal components with *snpgdsPCA* using all 4 autosomes. For subsequent analyses, we repeated the LD pruning within subsets of the data (North America only, or Carter Mountain, Virginia, only). We also calculated principal components using individual chromosomes without removing putative inversions; for the X chromosome, only females were used in the analysis. The single fly collected in Kenya in 2018 (Comeault et al. 2021) was an extreme outlier in the preliminary PCA and was excluded. We conducted the initial analysis using all SNPs that met the filtering criteria and then conducted a second analysis using SNPs that were masked for potential inversion regions as described above. We conducted discriminant analysis of principal components (DAPC) on 5 populations (Florida and 4 years from Carter Mountain) using the *dapc* function in package *adegenet* 2.1.10 (Jombart 2008; Jombart and Ahmed 2011). We excluded inversion regions and used 4 principal components in the DAPC analysis (number of groups − 1) following the guidelines from Thia (2023).

We used *Plink* v. 1.9 (Purcell et al. 2007; Chang et al. 2015) to LD prune VCF files with parameters (*−indep-pairwise 1000 50 0.2*) and used *ADMIXTURE* v. 1.3.0 (Alexander and Lange 2011) to evaluate population structure. Using whole genome data not masked for inversions, we calculated admixture for each chromosome separately. For the X chromosome, only females were used. We tested up to *k* = 10 genetic clusters and used cross-validation analysis to choose the optimal *k* for each chromosome separately. We also ran ADMIXTURE using all non-inversion autosomal SNPs together.

We calculated *F*_ST_ between Virginia samples and those from Florida or Africa using the *snpgdsFST* function in *SNPRelate* for all SNPs with a minor allele count > 3. For the X chromosome, only females were used in *F*_ST_ calculations. We used the same function to calculate genome-wide, pairwise *F*_ST_ between all Virginia collections using autosomal SNPs with putative inversions masked. To quantify relatedness between individuals, we used the function *snpgdsibdKING* in *SNPRelate* to determine the kinship coefficients and probability of zero identity by descent for pairs of individuals using autosomal SNPs with putative inversions masked. We used thresholds established in Thornton et al. (2012) to classify relatedness between individuals. We used the *–het* function in *vcftools* v. 0.1.17 to calculate the inbreeding coefficient for each individual using non-inversion SNPs.

We generated phylogenetic trees using *Treemix* (Pickrell and Pritchard 2012). We generated a vcf masked for inversions that contained only Carter Mountain and Florida samples. We LD-pruned this vcf in *PLINK* v. 1.9 with *--indep-pairwise 100 10 0.2* and prepared *Treemix* input files with custom scripts. We then ran 100 bootstrap replicates of *Treemix* with zero migration edges, Florida as the outgroup, and arguments *–bootstrap -k 500.* We used *PHYLIP* v. 3.696 (Felsenstein 2005) to generate a consensus tree and support for each node. We plotted a representative output in R using scripts from https://github.com/andrewparkermorgan/popcorn/.

### Estimation of historic population sizes

We used *smc*++ v. 1.15.4 (Terhorst et al. 2017) to estimate historic population sizes for several subpopulations of individuals using chromosome 4 genotypes since this chromosome lacked evidence of large structural rearrangements. We used individuals from each African location and used the earliest sampling available for each year and the Virginia orchard. We used *vcf2smc* to prepare the input files for each autosome separately. We assigned each individual as the “distinguished individual” and ran the analysis using all possible combinations of distinguished individual as described in (Bemmels et al. 2021). We used cross-validation to estimate final model parameters with the option (*-cv –folds = number of individuals)*. We assumed a generation time of 0.08 years (∼12 generations per year) based on Nava et al. (2007), which assumes year-round reproduction in tropical regions. We note that for Virginia populations experiencing temperate conditions in recent years, 12 generations per year is likely an overestimate due to the shortened breeding season.

### Selection scan

We used *WhatsHap* v. 1.7 (Patterson et al. 2015) to perform read-based phasing of the full vcf including singletons. To polarize the vcf for the genome-wide selection scan relative to the invasion, we reassigned the reference allele of the phased vcf as the allele that was most common across all African individuals sequenced in previous studies. We calculated allele frequencies using all African samples in *SNPRelate*, then used *vcf-info-annotator* (https://vatools.readthedocs.io/en/latest/index.html) to assign the “ancestral” allele in the INFO column. Lastly, we used *bcftools* v. 1.13 (Danecek et al. 2021) to make simplified vcfs containing only the GT and AA fields for each chromosome separately.

We used the R package *rehh* v. 3.2.2 (Gautier and Vitalis 2012) to conduct the selection scan using integrated haplotype homozygosity score (IHS). The test measures the decay of haplotype homozygosity to look for long, shared haplotypes that are signatures of selective sweeps when a single haplotype rises to high frequency without being eliminated by recombination (Sabeti et al. 2002; Voight et al. 2006). We used all flies from Virginia and conducted the scan using phased, polarized vcfs for each individual chromosome. We used the *haplo2hh*, *scan,* and *ihh2ihs* functions to implement the scan. For the X chromosome, we only used a single haplotype for each male in the dataset to avoid double-counting haploid genotypes.

We also used *BayPass* v. 2.41 (Gautier 2015) to calculate signals of selection comparing flies from Virginia to those from Florida and Africa while accounting for population structure via the XtX statistic. For this analysis, we used only females so that a single analysis could be conducted across all chromosomes. We created separate vcf files for each population and prepared *BayPass* input files using *vcftools –counts2*. We pruned these files for LD in *Plink* 1.9 using *--indep-pairwise 50 5 0.2.* We then ran *BayPass* according to the user manual and modified code from (Whiting et al. 2021) to generate a core model and covariance matrix and final XtX values. We used 10,000 simulated SNPs to define XtX outliers.

To identify shared signals across the *F*_ST_, XtX, and IHS selection/differentiation analyses, we used a window-based approach. We divided the genome into 1 kb windows with a 500 bp step size and identified all windows that contained at least one SNP that fell in the top 99% of SNPs for the *F*_ST_, XtX, and IHS tests.

### Genetic diversity statistics

Because we obtained variable sequencing coverage within and across populations (Supplementary Fig. 2), we used software designed for low coverage and missing data to analyze population genetic statistics in genomic windows. We used *pixy* v. 1.2.5 (Korunes and Samuk 2021) to calculate Pi, *F*_ST_, and *D*_XY_ in 5 kb windows. Samples were grouped by collection location; only females were used for the analysis of the X chromosome. We used *ANGSD* v. 0.941 (Korneliussen et al. 2014) to calculate Tajima's D. We first calculated genotype likelihoods from the bam files using arguments *-doSaf* and *-GL*. We then calculated Tajima's D and theta using the folded site frequency spectrum across 5 kb windows with 5 kb steps as described in *ANGSD* documentation using only female samples.

### Data management and plotting

We used the R packages *foreach* (Microsoft and Weston 2017) and *data.table* (Dowle and Srinivasan 2019) for data management and manipulation and used *ggplot2* (Wickham 2016) for all plotting. The *ggpubfigs* (Steenwyk and Rokas 2021) and *viridis* (Garnier 2018) packages were used for color palettes.

## Results and discussion

### Genome assembly and annotation

High-quality genome assemblies and annotations are a critical component of tracking and controlling invasive species and understanding their potential for evolution in invaded ranges (Matheson and McGaughran 2022). We conducted Hi-C-based scaffolding of a previously sequenced *Z. indianus* genome (Kim et al. 2021) to achieve a chromosome-level assembly. There were 1,014 scaffolds with an N50 of 26.6 Mb, an improvement from an N50 of 4.1 to 6.8 Mb in previous assemblies (Kim et al. 2021). The 5 main chromosomes (Supplementary Fig. 1, named in order of size from largest to smallest) varied in length from 25.7 to 32.3 Mb (total length of 5 main scaffolds = 146,062,119 bp), in agreement with *Z. indianus* karyotyping (Gupta and Kumar 1987; Campos et al. 2007). Chromosome 3 was identified as the X chromosome using sequencing coverage of known-sex individuals (Supplementary Figs. 3 and 4). See Supplementary Table 2 for assignment of *Z. indianus* chromosomes to Muller elements based on alignment to the *D. melanogaster* genome (Supplementary Fig. 5).

The annotation using RNAseq from larvae, pupae, and adults predicted 13,162 transcripts and 13,075 proteins, with 93% of 255 benchmarking universal single-copy orthologs (BUSCO) genes (Simão et al. 2015) identified as complete and an additional 1.2% of BUSCO genes identified as fragmented. This transcriptome-based completeness estimate is lower than the genome-based estimate of 99% complete (Kim et al. 2021) but is in line with other arthropod genomes (Feron and Waterhouse 2022). Within the 5 main chromosomes, 24.6% of sequences were repetitive; within the entire assembly, including all smaller scaffolds, 41% were repetitive. The 5 main chromosomes contain 11,327 predicted genes (87% of all predicted genes), including 99.5% of all complete BUSCO genes. This improved genome resource will be valuable for future evolutionary studies *of Z. indianus*, which is becoming an increasingly problematic pest in some regions of the world (Allori Stazzonelli et al. 2023).

### Limited spatial or temporal population structure in North American *Z. indianus*

To study spatial and temporal patterns of genetic variation in the seasonally repeated invasion of *Z. indianus*, we resequenced ∼220 individuals collected from 2 orchards in Virginia (Carter Mountain Orchard and Hanover Peach Orchard) from 2017 to 2020, as well as one population collected from Coral Gables, Florida, in 2019. Because temperate locations such as Virginia are thought to be recolonized by southern populations of *Z. indianus* each year (Pfeiffer et al. 2019; Rakes et al. 2023), we sampled both early in the season (∼July to August) and late in the season (∼October to November) in each year to capture the founding event, population expansion, and potential adaptation to the temperate environment.

We were first interested in studying geographic and temporal variation in population structure in North American populations of *Z. indianus*. For this analysis, we incorporated previous sequencing data from the Western Hemisphere and Africa (Supplementary Table 1 and Comeault et al. 2020). While previous studies have shown limited structure within North America (Comeault et al. 2020, 2021), we wanted to test for structure using deeper sampling within introduced locations and with greater temporal resolution across the *Z. indianus* growing season (Rakes et al. 2023). Our initial analyses of population structure using PCA of the whole genome (Supplementary Fig. 6a) and individual chromosomes (Supplementary Fig. 7a), as well as *ADMIXTURE* (Alexander and Lange 2011) analysis of individual chromosomes (Supplementary Fig. 8) confirmed previous results that Western Hemisphere samples are genetically distinct from African samples. To focus on potential structure within invasive North American samples, we excluded the African samples and recalculated principal components. North American samples fell into three large groups when considering PC1 and PC2 (diagonal bands in Supplementary Fig. 6b), but these clusters generally did not correspond to sampling locations. In single-chromosome PCA, North American samples formed clusters that did not correspond to geographic locations for chromosomes 1, 2, 3, and 5; such patterns are potentially indicative of inversions that influence genotypes of many SNPs simultaneously (Li and Ralph 2019). This pattern was upheld in the *ADMIXTURE* analysis; notably, for chromosomes 1, 2, and 5, many individuals showed ∼50% ancestry assignment to different clusters, which could reflect genotypes for large structural rearrangements. These findings led to further investigations of potential structural polymorphisms (described below) and the masking of SNPs potentially located in large chromosomal rearrangements to examine population structure.

**Fig. 1.**
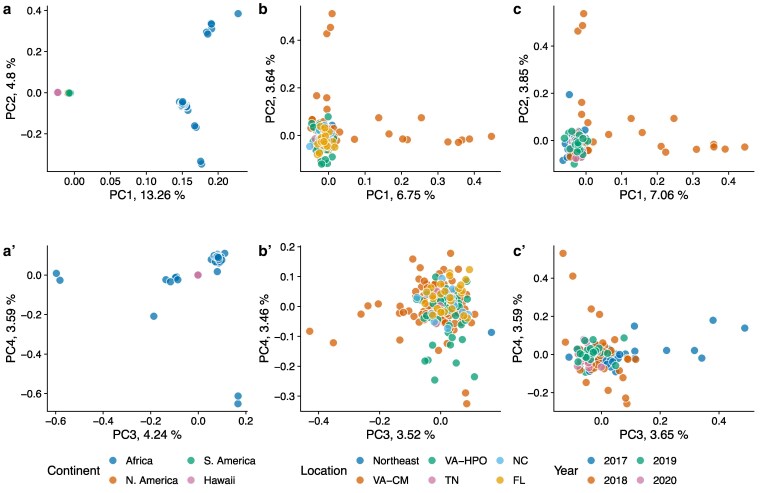
Principal component analysis of *Z. indianus* populations using autosomal SNPs outside of putative inversions reveals a lack of spatial or temporal population structure in invasive populations. Top row shows PC1 and PC2; bottom row shows PC3 and PC4. a–a′) All individuals (*n* = 266), color coded by continent/locale of collection. South America, North America, and Hawaiian samples are all tightly clustered such that individual points are not visible. b–b′). All North American individuals (*n* = 224), color coded by collection site; HPO and CM are 2 orchards in Virginia; Northeast refers to samples from NY, NJ, and PA. c–c′) All individuals from Carter Mountain, Virginia (*n* = 127), color coded by year of collection. For each analysis, only the individuals shown in the plot were included in the PC calculation.

After removing regions of the genome that were potentially part of inversions (see below), PC1 still separated Western Hemisphere and African samples (Fig. 1a), and North American samples remained tightly grouped for PCs 1 to 4, indicating most genetic variation is found within African samples. Analysis of only North American samples revealed little genome-wide separation of populations collected from different locations (Fig. 1b), though some Carter Mountain, VA, individuals were outliers from the main cluster of points. Many invasive species evolve complex population structures in the invaded range due to a combination of bottlenecks, founder effects, and rapid local adaptation (Koch et al. 2020; Atsawawaranunt et al. 2023; García-Escudero et al. 2024). On the other hand, some invasive species have more homogenous populations across widespread invaded ranges in eastern North America (Friedline et al. 2019; Barrett et al. 2023). A high rate of migration between orchards (occurring naturally or due to human-mediated transport) or large founding population sizes could result in a lack of geographic differentiation between populations.

We next hypothesized that founder effects during each recolonization event might lead to unique genetic compositions of temperate populations sampled in different years (Uller and Leimu 2011). We calculated principal components using only samples collected from Carter Mountain, Virginia, between 2017 and 2020. Surprisingly, in these samples, we saw no evidence of population structure between years (Fig. 1c), apart from some samples from 2018 that were divergent from the rest; we investigate potential causes of this pattern below. These data suggest that the founding fly populations in Virginia are relatively homogeneous each year at a genome-wide scale, but some years may contain genetically divergent individuals. This result is consistent with the lack of spatial population structure and likewise could indicate large founding populations or ongoing migration. Alternatively, the Virginia populations could be permanently established with little genetic differentiation year-to-year, though this possibility is not supported by field data from temperate locations (Rakes et al. 2023; Kohlmeier and Kohlmeier 2025).

**Fig. 2.**
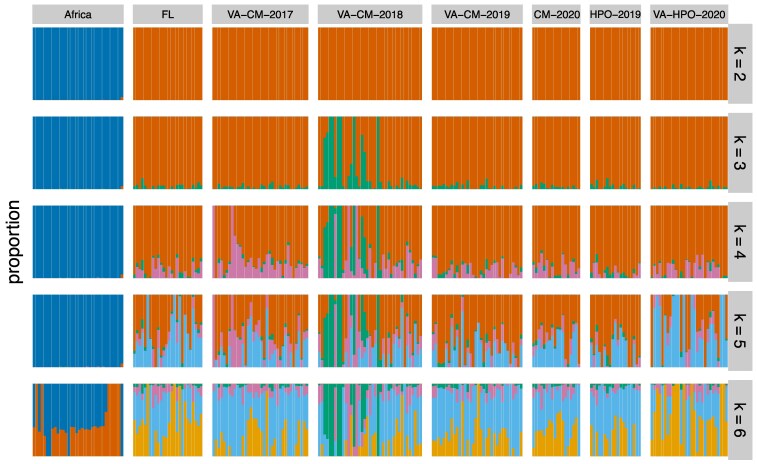
Admixture analysis of *Z. indianus* from different locations. Each column is an individual, and colors represent assignment to distinct genetic clusters based on all autosomal SNPs outside of inversions. Each row represents a different number of ancestral genetic clusters (*k* = 2 to 6). FL, Coral Gables, Florida; VA-HPO, Hanover Peach Orchard, Virginia; VA-CM, Carter Mountain, Virginia. African sequences combine 5 geographic locations from Comeault et al. (2020, 2021). Cross-validation analysis supported *k* = 2 (top row) as the most likely number of ancestral groups.

*ADMIXTURE* revealed 2 ancestral clusters for global *Z. indianus* populations, separating Africa from North America (Fig. 2). Adding increasing numbers of ancestral groups revealed genetic differentiation within North American samples, but this differentiation did not correspond to location or year of collection. Notably, with *k* = 3, some individuals from Carter Mountain in 2018 separated from the other North American samples; these samples correspond to those that were outliers in the PCA. Collectively, our results are consistent with previous findings that African populations are distinct from those in the Western Hemisphere, and we find little evidence of genome-wide population structure over space or time within North American samples.

### Structural polymorphism

The clustering of samples in the single-chromosome PCA (Supplementary Fig. 7), combined with many individuals showing ∼50% assignment to genetic clusters within individual chromosomes (Supplementary Fig. 8), suggested that large structural variants may be segregating in *Z. indianus* (Li and Ralph 2019; Nowling et al. 2020). To look for evidence of structural variants via depressed recombination rates, we examined LD from 4,000 randomly sampled SNPs on each chromosome. In North American samples, we discovered large blocks of LD spanning substantial portions of chromosomes 1, 2, 3, and 5 (Supplementary Fig. 9), potentially indicative of inversions (Fang et al. 2012; da Silva et al. 2019). However, there was no evidence of long-distance LD in these regions in the African samples (Supplementary Fig. 9). These results suggest inversions on chromosomes 1, 2, 3, and 5 are segregating in North America but are relatively rare in Africa. To approximately define the boundaries of these inversions, we calculated LD for random pairs of SNPs that were 100 to 500 kb apart on each chromosome. This analysis similarly identified large regions containing elevated long-distance LD on chromosomes 1, 2, 3, and 5 (Supplementary Fig. 10a). We compared the results of the LD analysis to potential inversions called using paired-end data by *smoove* but found little correspondence (Supplementary Fig. 10b, compare gray boxes to yellow boxes). As such, we used the LD-based data to mask potential inversion regions for population structure analyses described above.

Given the relative chromosome sizes in the genome assembly, the linkage blocks on chromosomes 2 and 5 likely correspond to the previously described inversions *In(V)B* and *In(II)*A, respectively (Ananina et al. 2007). Since chromosome 1 is the longest chromosome in our assembly, the linkage likely correspond to the complex *In(IV)EF* polymorphism, made up of 2 overlapping inversions (Ananina et al. 2007); different genotypic combinations of 2 inversions could explain the 6 distinct clusters seen in the chromosome 1 PCA (Supplementary Fig. 7b). The X chromosome has 3 described inversions in *Z. indianus* (Ananina et al. 2007), which may explain to the complex pattern of linkage observed in North American samples on this chromosome (Supplementary Figs. 9 and 10) and the clustering of North American samples in the chromosome 3 PCA (Supplementary Fig. 6b, c). Major chromosomal polymorphisms are known to be important for local adaptation and phenotypic divergence in a wide variety of species (Joron et al. 2011; Küpper et al. 2016; Lee et al. 2016; Huang et al. 2020; Nunez et al. 2024), including inversions that facilitate invasive phenotypes (Galludo et al. 2018; Tepolt and Palumbi 2020; Tepolt et al. 2022; Ma et al. 2024). These inversions may be new to North America, or they may have been present at low frequency in the bottlenecked population that founded *Z. indianus* populations in the Western Hemisphere but then experienced subsequent selection in the invaded range. Alternatively, they may have arisen in a currently unsequenced population and then been introduced to the Western Hemisphere. Further characterization of these inversions through additional sequencing and phenotypic characterization to determine whether they influence *Z. indianus* survival or fitness in the invaded range will be a rich area for future studies.

### Recolonization, bottlenecks, and seasonal dynamics in *Z. indianus*

**Fig. 3.**
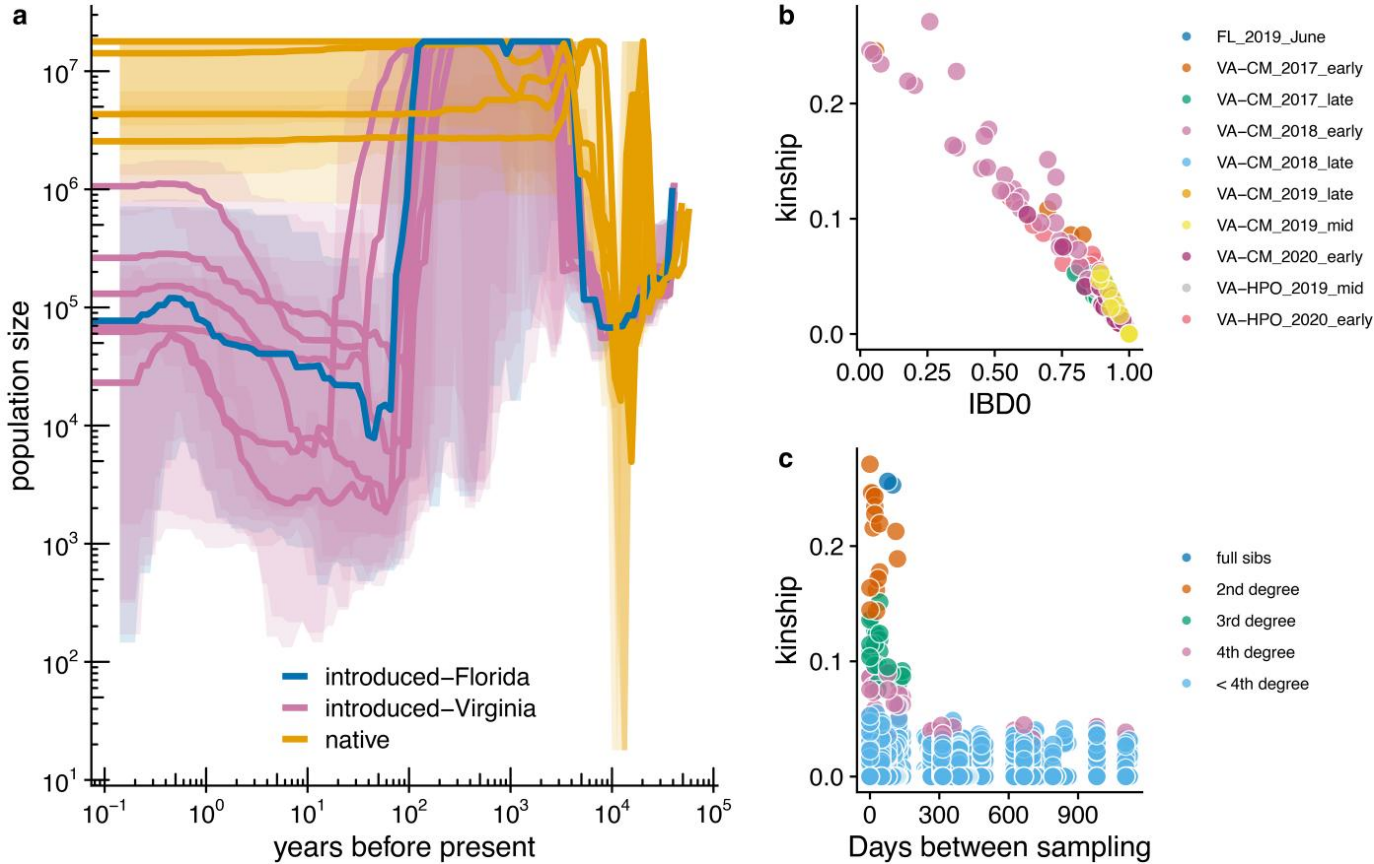
Demographic effects of bottlenecks in *Z. indianus* populations. a) Population history reconstruction with *smc++* using genotypes from chromosome 4, which lacks inversions. Introduced-Virginia flies were collected in the early–mid season (June to September) from 2 Virginia orchards between 2017 and 2020 (*n* = 6 populations grouped by orchard and year). Native populations are 5 distinct African populations (Kenya, Zambia, Senegal-Forest, Senegal-Desert, and Sao Tome [Comeault et al. 2020]). Shaded ribbons show minimum and maximum population sizes estimated in cross-validation analysis. b) Kinship and probability of zero identity by descent for pairs of individual flies from the same collection location and season within North America calculated with non-inversion autosomal SNPs. c) Kinship coefficients for pairs of individual flies collected at Carter Mountain Orchard, Virginia, as a function of the number of days between sampling. Relatedness was assigned according to thresholds from Thornton et al. (2012).

Invasive species typically experience a genetic bottleneck due to small founding population sizes (Barrett 2015; Estoup et al. 2016). We hypothesized that North American populations would show a reduced effective population size (*N*_e_) relative to African populations, and that Virginia populations would show a further, more recent reduction in *N*_e_ relative to the Florida population as the result of a secondary population bottleneck upon temperate recolonization. We estimated historic population sizes using *smc++* (Terhorst et al. 2017) using data only from chromosome 4, which lacks large inversions (Fig. 3a). Our prediction was correct with respect to Africa vs North America: African populations show historical fluctuations but population sizes typically in the range of ∼10^4^ to −10^7^ individuals (10^6^ to −10^7^ for the past ∼1,000 years). However, introduced populations in North America demonstrated a dramatic reduction in population size in the past ∼100 years, perhaps reflecting a bottleneck following colonization of Brazil in the late 1990s (Yassin et al. 2008) and consistent with the previously described loss of genetic diversity in invasive populations (Comeault et al. 2020). This contraction is followed by a rebound as introduced populations expanded over the past several decades. Overall, the ancestral population sizes for Virginia and Florida were quite similar, with overlapping ranges of the estimates, and our prediction of reduced recent population sizes in Virginia relative to Florida was not well-supported. Given our limited sample sizes and potential differences in the number of generations per year in temperate and subtropical environments, detecting fine-scale differences in very recent population fluctuations may be beyond the detection ability of the software; *smc++* becomes less accurate at timescales less than ∼333 generations (∼26 years for year-round populations of *Z. indianus*) (Patton et al. 2019). Virginia populations could be colonized by a large number of individuals, or they may represent admixed populations reflecting individuals from multiple sources, producing larger effective population sizes than would otherwise be expected if recolonization occurs from a single source population undergoing a bottleneck. Admixture and gene flow are important factors fueling genetic diversity and invasiveness in introduced species (McGaughran et al. 2024) and could potentially contribute to *Z. indianus*' local success following each recolonization event.

We additionally tested for bottlenecks by looking for the presence of relatives within our samples, which might be a product of small founding populations. Using 2 measures of genetic similarity, we discovered many pairs of related flies in our dataset (Fig. 3b). Most dramatically, many flies collected in 2018 appeared to be close relatives (Supplementary Fig. 11), consistent with the separation of some 2018 samples in the PC and *ADMIXTURE* analyses (Figs. 1c and 2). In collections from late July and early August 2018, 26 pairs of close relatives involving 13 individual flies were collected. Of those, 21 pairs of relatives were collected on different days, suggesting the relatedness was not solely a sampling artifact due to collecting relatives in the same microhabitat of the orchard. The effect of this apparent bottleneck was sometimes retained throughout the growing season, as a pair of full sibs was sampled 77 d apart in 2018, 2 pairs of second-degree relatives were sampled over 110 d apart in 2018, and 2 pairs of third-degree relatives were sampled 140 d apart in 2017 (Fig. 3c). Given that *Z. indianus* are collected in small numbers early in the season (Rakes et al. 2023) and 2017 and 2018 had particularly early captures (Table 1), we thought a small founding population size followed by inbreeding could produce individuals sampled distantly in time that still show close genetic similarity. However, we did not find any significant difference in inbreeding coefficients between flies sampled at different time points at Carter Mountain (ANOVA, *F*(6,120) = 1.79, *P* = 0.106). Instead, flies may live for a relatively long time or have slower generations in the wild, allowing us to capture close relatives separated by longer time periods.

A founder effect could generate temporal population structure by creating populations that were more similar within a year than between years, creating a positive relationship between *F*_ST_ and the elapsed time between collections (Bergland et al. 2014). We tested this prediction with samples collected from Carter Mountain, Virginia, over 4 years and found a weak, positive correlation between *F*_ST_ and the time between sample collections for comparisons that excluded the unusual 2018 collection containing many relatives (linear model, *df* = 19, *R*^2^ = 0.423, *P* = 0.009, Supplementary Fig. 12). This finding is consistent with trends observed in *D. melanogaster*, which experiences a strong overwintering bottleneck and shows temporal patterns of differentiation (Bergland et al. 2014; Nunez et al. 2024). Since this finding could be caused by an overwintering bottleneck or a recolonization bottleneck, we further investigated population relationships over time.

**Fig. 4.**
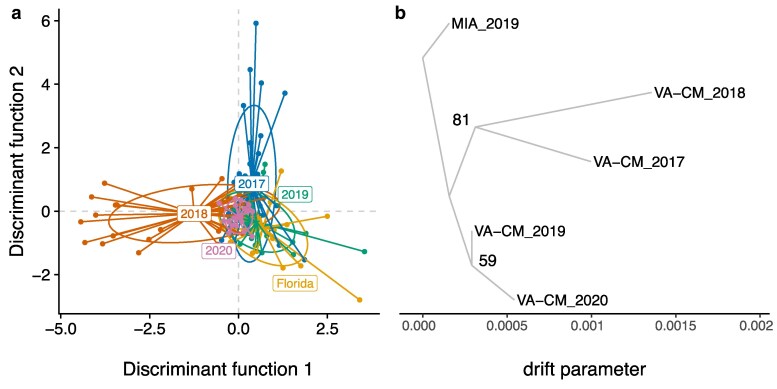
DAPC and *Treemix* analyses suggest *Z. indianus* recolonizes Virginia each year. All analyses were conducted with autosomal SNPs outside of inversions. a) DAPC of samples from Florida and 4 years of sampling from Carter Mountain, Virginia using 4 principal components. b) Results of a single *Treemix* run representative of the consensus tree topology from *PHYLIP*; bootstrap support values from 100 trees are given at nodes.

We used DAPC and phylogenetic trees to look at relationships between flies captured at Carter Mountain, Virginia, over time relative to the population from Florida. In North America, *Z. indianus* was first found in Florida and was later found northwards (van der Linde et al. 2006; Pfeiffer et al. 2019). If flies are permanently established in Virginia, we predict that the Virginia populations would become progressively more different from Florida over time in a “stepping-stone” like pattern as each population evolves independently. If flies recolonize each year with small founding population sizes, we expect populations to be more similar to Florida in some years than others due to chance. The predictions of recolonization were supported by the DAPC analysis, which showed that the 2019 and 2020 collections were almost entirely overlapping with those from Florida, while the 2017 and 2018 collections showed a mixture of overlapping and more divergent samples (Fig. 4a). Overall, there was no clear pattern of temporal differentiation, and the later samples were more similar to Florida than the earlier samples were. We also used *Treemix* (Pickrell and Pritchard 2012) and *PHYLIP* (Felsenstein 2005) to build a consensus phylogenetic tree from 100 independent bootstraps of the same dataset. The topology of the consensus tree (Fig. 4b) was not consistent with stepwise divergence of the populations over time; the 2 earlier timepoints formed a clade and the 2 later timepoints formed a clade, though the node for the 2019 to 2020 divergence was not well supported. The drift parameter was greater in 2017 and 2018 than it was 2019 and 2020, suggesting that populations collected later in time were more similar to Florida than those collected earlier in time. This result is consistent with a recolonization model in which the founding population is randomly more representative of the source population in some years than others, with smaller and earlier founding events in 2017 and 2018 potentially leading to greater genetic drift. Additional demographic simulations will likely be required to more definitively determine the colonization dynamics of *Z. indianus*.

### Signals of recent selection in *Z. indianus* near potential pesticide resistance genes

**Fig. 5.**
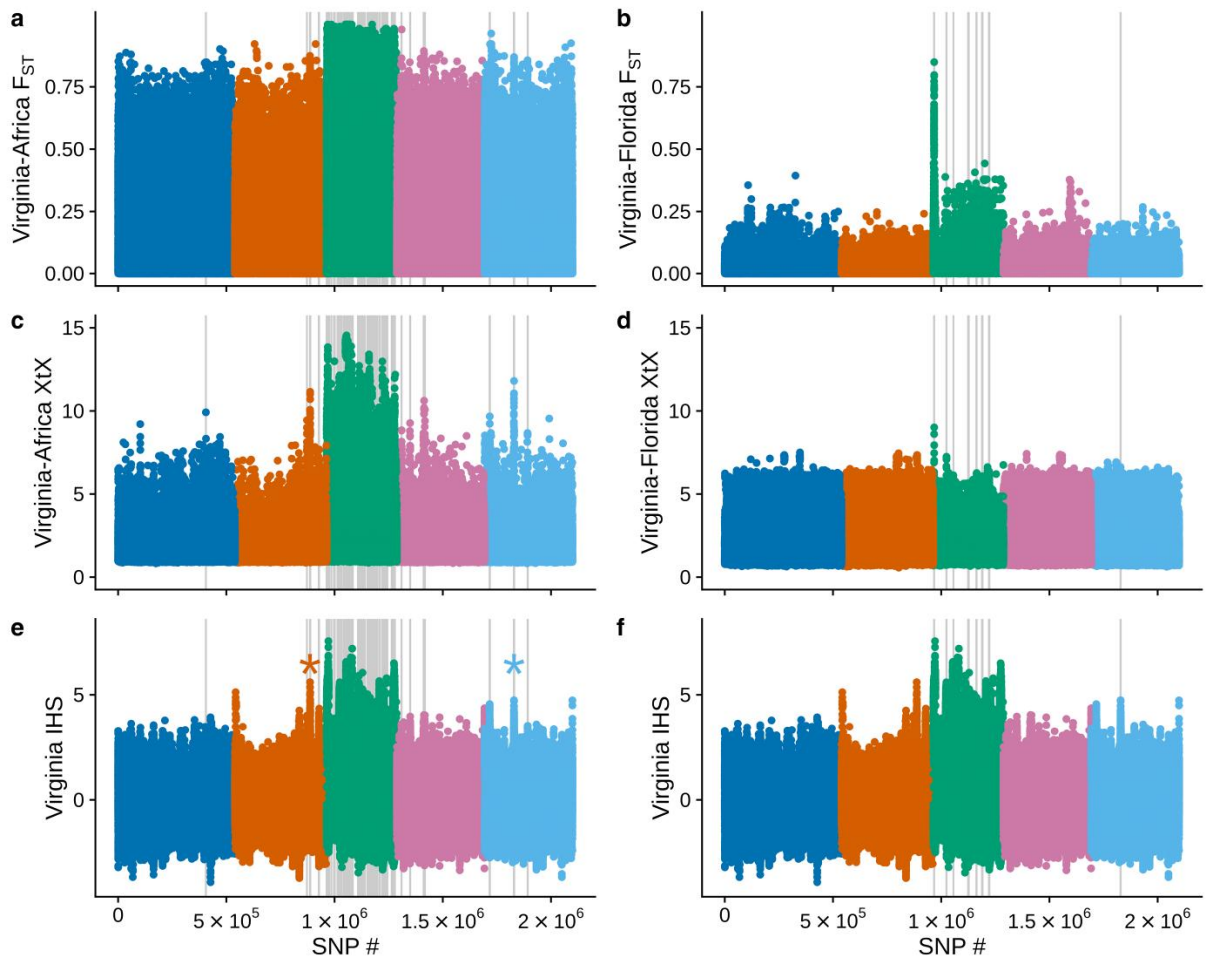
Signals of differentiation and selection in temperate *Z. indianus* populations. a) and b) Genome-wide *F*_ST_ comparing individual flies sampled in Virginia (*n* = 175) to all flies sampled in Africa (a, *n* = 34) or Florida (b, *n* = 26); colors indicate chromosomes 1 to 5 in order. Only females were used for the X chromosome analysis (chromosome 3, green). c) and d) BayPass XtX statistic for comparisons of females from Virginia and Africa (c) and Virginia and Florida (d). e) and f) IHS testing for long, shared haplotypes in all flies collected in Virginia. The IHS datapoints in panels (e) and (f) are the same but presented twice to show alignments with each analysis above. Vertical lines indicate locations of 1 kb windows that contain *F*_ST_, XtX, and IHS scores in the top 1% genome-wide; windows were calculated separately for the Virginia-Africa and Virginia-Florida comparisons. Asterisks in panel (e) indicate the shared peaks on chromosomes 2 (left asterisk) and 5 (right asterisk) discussed in the text and examined in Fig. 6.

We tested for signals of recent natural selection in temperate *Z. indianus* populations from Virginia. We compared Virginia populations to those from Africa to test for broader invasive-native population differentiation and compared Virginia to Florida to look for potentially more recent temperate adaptation. We measured 3 statistics: SNP-level *F*_ST_ comparing all flies collected in Virginia with those from Florida or Africa; the XtX statistic from *BayPass* (Gautier 2015) to test for selection in the presence of population structure; and IHS to look for long, shared haplotypes that are signatures of recent selective sweeps when a single favorable haplotype rises to high frequency (Sabeti et al. 2007). We note that our approach would not detect sweeps involving multiple alleles from standing variation (soft sweeps; Messer and Petrov 2013; Garud et al. 2015), which could be an important potential component of *Z. indianus* evolution given the high levels of genetic diversity found in invasive populations (Avalos et al. 2017; Comeault et al. 2020). To compare results across tests, we identified for 1 kb windows containing SNPs falling within the top 1% of all 3 tests. As expected given the high degree of population structure, SNPs with high *F*_ST_ between Africa and Virginia were widespread across all chromosomes, and *F*_ST_ was particularly high on the X chromosome (Fig. 5a). This observation is in line with the findings of Comeault et al. (2021), who showed that many X-linked scaffolds showed signs of selection in invasive populations, and we suggest this pattern is likely related to the smaller effective population size for X chromosomes (Ellegren 2009) and the presence of several inversions on this chromosome (this study; Ananina et al. 2007). After accounting for population structure with *BayPass*, the signal remained high throughout most of the X chromosome, but several peaks of potential selection were resolved on the autosomes, including prominent peaks on chromosomes 2 and 5 (Fig. 5c). These peaks corresponded to windows with long, shared haplotypes in Virginia populations (high IHS scores, Fig. 5e). The complex population structure and broadly elevated signals of divergence and selection on the X chromosome made it difficult to investigate candidate loci further. In contrast, only a small number of autosomal windows were found to contain elevated signals for all 3 tests, so we focused on the 2 autosomal windows that had the most pronounced signals for the *BayPass* and IHS analysis: Chromosome 2 at ∼26.6 Mb and chromosome 5 at ∼7.9 Mb (asterisks in Fig. 5e).

We examined the genes annotated in each of these outlier regions and found that both regions included genes in the cytochrome P450 pathway, which is involved in detoxifying endogenous and exogenous compounds including synthetic pesticides (Scott et al. 1998). The IHS peak on chromosome 2 is found near the *Z. indianus* homolog of *D. melanogaster cpr*, a cytochrome P450 reductase essential for electron transfer in the detoxification activities of cytochrome P450 proteins (Hannemann et al. 2007). Decreased *cpr* expression has been linked to susceptibility to pesticides in a variety of insect pests and disease vectors (Lycett et al. 2006; Wang et al. 2016, 2025; He et al. 2020; Shi et al. 2021). A proline-to-leucine polymorphism in *cpr* has an XtX score of 7.63, is not found in Africa, and has a frequency of 0.57 in Virginia. Two other SNPs upstream of *cpr* also have large allele frequency differences between Virginia and Africa, offering interesting candidates for further study that might influence gene regulation.

The IHS peak on chromosome 5 is near a cluster of 5 cytochrome P450 genes. Although additional work will be required to resolve CYP gene orthologs and paralogs in *Z. indianus*, the cluster contains several *Cyp6* genes. One of the largest signals of selective sweeps in the *D. melanogaster* genome is found in *Cyp6g1* (Garud et al. 2015); alleles of *Cyp6g1* are associated with resistance to dichloro-diphenyl-trichloroethane (DDT) and neonicotinoids (Daborn et al. 2001; Schmidt et al. 2010). Therefore, mutations in *Z. indianus Cyp6* genes might also be involved in insecticide resistance. For example, in this region, missense variants in *Cyp6a9* and *Cyp317a1* have large allele frequency differences between Virginia and Africa; *Cyp317a1* was associated with permethrin resistance in the Drosophila Genetic Reference Panel (Battlay et al. 2018). *Z. indianus* is a substantial crop pest in Brazil, and several studies have indicated Brazilian *Z. indianus* populations may have evolved some degree of pesticide resistance in response to the use of organophosphorous compounds to protect fig crops (Galego and Carareto 2010; de Oliveira Rios et al. 2024 ). Although *Z. indianus* is not currently categorized as a pest in the United States (Animal and Plant Health Inspection Service 2025) and is not an active target of management in eastern US orchards, many orchards use insecticides to control *D. suzukii* (Tait et al. 2021) and other arthropod pests. Controls for *D. suzukii* are often applied at the time of fruit ripening, which means that other species such as *Z. indianus* that are present during and after ripening may also be impacted by chemical control. Chemicals applied closer to the time of ripening may be more likely to remain on the overripe and decomposing fruit typically used as a breeding substrate for *Z. indianus.* Further investigation will be required to determine if mutations at these loci cause differences in pesticide resistance between African and North American flies. Insecticide resistance is a major challenge in the control of invasive insect pests (Siddiqui et al. 2023 ), and characterizing resistance may be important for future control of *Z. indianus*.

**Fig. 6.**
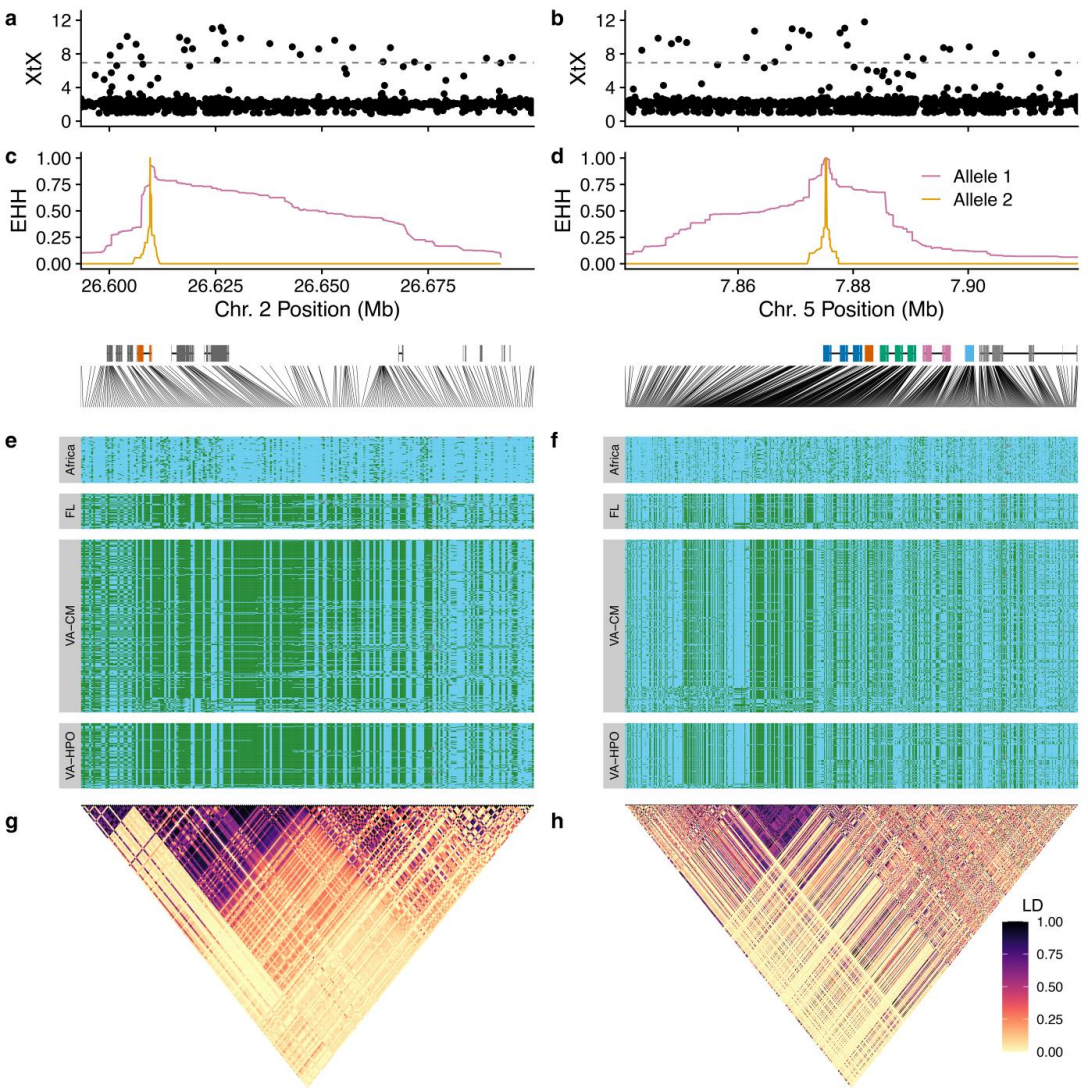
Haplotypes near potential pesticide resistance genes with signals of selection and differentiation. a) and b) *BayPass* XtX statistics for individual SNPs comparing Africa and Virginia populations (see Fig. 5a) for peaks on chromosomes 2 (a) and 5 (b). Horizontal lines indicate the 99.9% quantile of XtX scores from 10,000 simulated SNPs. c) and d) EHH for the SNP with highest IHS score at each locus (chr. 2: 26,609,601 and chr. 5: 7,875,359). The locations of annotated genes are shown below the *x*-axis. In panel (c) the gene highlighted in orange is *Cytochrome P450 reductase* (*cpr*). In (d) the genes highlighted in color are a cluster of 5 *Cytochrome P450* genes (predicted as *Cyp6a14*, *Cyp6a22*, *Cpy317a1*, *Cyp6a13*, and *Cyp6a9* from left to right). Lines connect SNP order for panels below to physical locations on chromosomes. e) and f) Haplotypes at each locus: each horizontal row shows genotypes for a single haploid chromosome phased with read-backed phasing. Light blue indicates the allele more common in African populations and dark green is the other allele. Missing genotypes are shown in gray. Panel (e) shows 223 SNPs and panel (f) shows 599 SNPs. g) and h) LD (*R*^2^) heatmaps for all SNPs for each haplotype demonstrate linkage in North American flies.

We confirmed the potential signal of selective sweeps by looking at haplotype structure in these 2 regions. Each region of high XtX score (Fig. 6a, b) was characterized by a broad peak of extended haplotype homozygosity (EHH) specific to the allele found in invasive populations (Fig. 6c, d), demonstrating that the candidate alleles have a slow decay of haplotype homozygosity. Visualization of the alleles found in each haplotype showed long haplotype blocks in North American populations that were not found in Africa (Fig. 6e, f); SNPs within these blocks demonstrate high LD in North American flies (Fig. 6g, h). Further examination revealed these haplotypes were found in Colombia, suggesting they may have originated after *Z. indianus* arrived in the Western Hemisphere but may predate *Z. indianus*'s arrival in North America. In invasive copepods, haplotypes under selection in the invasive range are ancestral polymorphisms under balancing selection in the native range (Stern and Lee 2020). A similar situation was found for a balanced inversion polymorphism that fuels invasion in invasive crabs (Tepolt and Palumbi 2020; Tepolt et al. 2022). However, ancestral polymorphisms selected in the invaded range do not appear to be the case in *Z. indianus*, as the haplotypes from North America were not found in African flies. These novel haplotypes could be new mutations or derived due to hybridization/introgression from another species or divergent population; hybridization can be an important evolutionary force in invasive species (Ellstrand and Schierenbeck 2000; Fournier and Aron 2021). The *Zaprionus* genus shows signals of historic introgression among several species, though *Z. indianus* was not directly implicated in a previous analysis (Suvorov et al. 2022). Therefore, 2 major haplotypes not found in Africa have been selected in Florida and Virginia populations, though the source of these haplotypes remains to be determined.

**Fig. 7.**
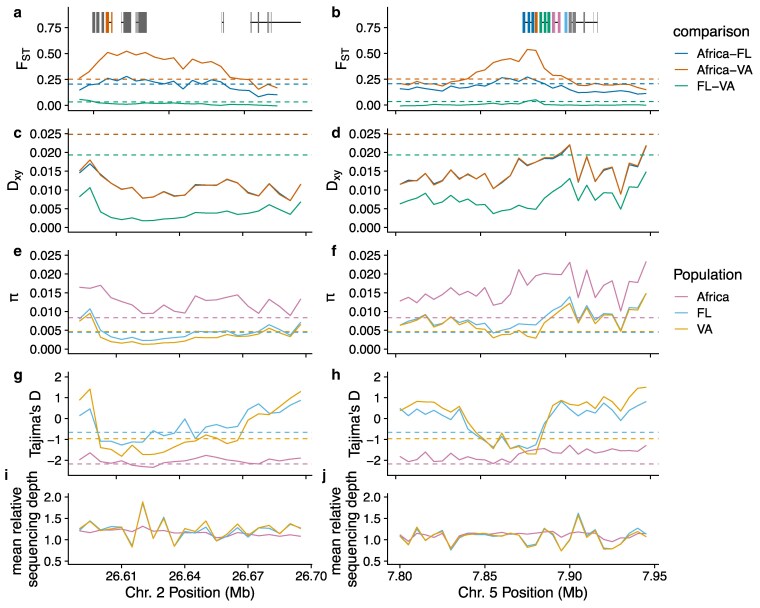
Population genetic statistics for the regions surrounding haplotypes on chromosomes 2 and 5. All statistics were calculated for 5 kb, nonoverlapping windows. Panels on left focus on the chromosome 2 region containing cpr, and the panels on right focus on the chromosome 5 region containing several *Cyp* genes. Gene locations are shown at the top using same color scheme as in Fig. 6. a) and b) *F*_ST_ comparing combinations of flies from Africa, Virginia (both focal orchards combined) and Florida. c) and d) Absolute nucleotide divergence (*D_xy_*) for the same comparisons. For (a) and (d), horizontal lines indicate 99th percentile of all autosomal windows. e) and f) Nucleotide diversity (π) for each population. g) and h) Tajima's D for the 3 populations. For (e)–(h), horizontal lines indicate bottom 1st percentile of all autosomal windows. i) and j) Average sequencing depth per window relative to the mean depth for the entire chromosome. Relative depths were averaged for all individuals in each population. See Supplementary Fig. 13 for whole-genome visualization of the same statistics.

To explore population genetic signals around these potential regions of selection, we broadly grouped flies into 3 populations—Africa, Florida, and Virginia—and calculated population genetic statistics in 5 kb nonoverlapping windows. *F*_ST_ between African and North American populations fell in the top 99% of all autosomal windows for both regions (Fig. 7a, b), suggesting a high degree of genetic divergence. However, absolute genetic divergence (*D_xy_*) between African and North American populations was not especially elevated in these windows (Fig. 7c, d). Nucleotide diversity (pi) was in the lowest 1% of all autosomal windows for North American samples (Fig. 7e, f), as predicted for a sweep that results in shared haplotypes across many individuals. Tajima's D was also negative and in the lowest 1% of all autosomal windows for North America, indicative of a recent sweep (Fig. 7g, h). Tajima's D was also negative in Africa for these regions, but was it negative across the entire genome (Supplementary Fig. 15), likely due to combining genetically disparate subpopulations into a single population for this analysis, producing an excess of rare variants. Relative sequencing depth showed fluctuations in North American samples that were not seen in Africa (Fig. 7h, i), suggesting copy number variants may exist in both regions. Collectively, these tests provide additional evidence that both regions may have been subject to recent selective sweeps.

For comparison, we also examined genetic diversity and population divergence across the genome (Supplementary Fig. 13) and observed that the X chromosome is an outlier in many regards. Divergence between African and North American samples is highest on the X chromosome (Supplementary Fig. 13a, b). As previously described (Comeault et al. 2020, 2021), the X chromosome has reduced genetic diversity relative to autosomes, especially in invaded populations (Supplementary Fig. 13c). X chromosomes have smaller effective population sizes in species with XY sex determination systems and often experience more extreme loss of genetic diversity upon population contraction (Ellegren 2009). Tajima's D is mostly positive in North American autosomes, indicative of a strong bottleneck in North American flies. However, Tajima's D fluctuates between strongly positive and strongly negative in North American populations along the X chromosome (Supplementary Fig. 13d). These findings, combined with many regions with high haplotype homozygosity on the X chromosome (Fig. 5e, f), suggest complex evolutionary dynamics on the X that warrant further investigation. Further global sampling and sequencing of X chromosomes with long reads to resolve inversion genotypes and CNVs may offer insight towards the potential role of X-linked variants in fueling the ongoing invasion of *Z. indianus*.

We lastly compared samples from Virginia and Florida to test for possible recent adaptation within the invasive range of *Z. indianus*. Virginia has a temperate, seasonal climate with a relatively limited variety of cultivated produce, and southern Florida is subtropical with an abundance and diversity of fruits throughout the year. Other factors such as diseases, insecticide use, and competing species may also differ widely between locales. In the absence of genome-wide population structure, genomic regions differentiated between these locations are candidates for local adaptation. We observed elevated *F*_ST_ throughout much of the X chromosome, with a pronounced peak at 690 kb (Fig. 5b); this peak was found near the most pronounced peaks in the XtX (Fig. 5d) and IHS (Fig. 5f) statistics. Examination of this region of the genome showed several long, shared haplotypes with high linkage in North American populations (Supplementary Fig. 14); in particular, one haplotype is nearly fixed in Virginia but is segregating in Florida. It is possible that this haplotype is associated with one of the structural variants on the X, and differences in inversion allele frequencies between Florida and Virginia could be driving these patterns. We examined population genetic signals in the region of highest *F*_ST_ (Supplementary Fig. 15) and determined that despite high *F*_ST_ from ∼550 to 650 kb (Supplementary Fig. 15a), *D_xy_* between Florida and Virginia was not high in this region, nor were nucleotide diversity (Supplementary Fig. 15c) or Tajima's D (Supplementary Fig. 15d) particularly low. In fact, immediately adjacent to the region of high FST was a region of nearly zero divergence between Florida and Virginia from ∼700 to 800 kb (Supplementary Fig. 15a, b). This region of low divergence had low nucleotide diversity (Supplementary Fig. 15c) and negative Tajima's D (Supplementary Fig. 15d), indicating it could be a sweep in North America. However, it was immediately adjacent to a region of irregular sequencing depth (Supplementary Fig. 15e), suggesting the signals we observed could be confounded by read mapping artifacts. This region contained a large repetitive element (Supplementary Fig. 16a), and sequencing depth was variable across populations (Supplementary Fig. 16b), suggesting structural variants such as duplications or deletions could be influencing mapping and population genetic statistics in this region. For example, at ∼610 kb, Florida samples had greater depth than Virginia, but from ∼625 to 650 kb, Virginia samples had greater depth than Florida or Africa. All samples had low coverage near the repetitive element. These findings suggest copy number variation near repetitive elements at these loci might contribute to the Florida–Virginia divergence; whether the divergence is an artifact due to sequence misalignments or due to real copy number variation will require further analysis with long-read sequencing. The region of highest divergence between Florida and Virginia contains ∼6 genes, including the gene *yin/opt1*, which is important for absorption of dietary peptides in *D. melanogaster* (Roman et al. 1998). If the Florida–Virginia divergence in this region is not a sequencing artifact, allelic differences within the invasive range in this gene could be involved in adaptation to new diets in new environments.

## Conclusions

In addition to posing economic, health, and environmental threats, invasive species also serve as outstanding models for studying rapid evolution in new environments. Here, we report an improved genome assembly and annotation for *Z. indianus*, an introduced drosophilid that is thought to repeatedly recolonize temperate environments each year and is a potential crop pest. We use it for a preliminary assessment of potential rapid evolution and genetic variation in the early stages of invasion. We show that recolonization is likely a stochastic process resulting in different evolutionary dynamics in different years, even within a single orchard. This finding demonstrates that broad sampling is important for invasive species that are repeatedly introduced or have multiple introduced populations that may undergo different evolutionary trajectories in different years or different locations. While some founding populations may be small, several population genetic patterns we observe could be explained by ongoing gene flow with the source population or between temperate populations following recolonization, suggesting gene flow that spreads and maintains favorable alleles could be an important component in *Z. indianus*'s widespread success, as it is for many invasive species (Díez-del-Molino et al. 2013; Medley et al. 2015; Arredondo et al. 2018). Demographic simulations and additional whole genome data will be required to better describe the recent histories of and potential gene flow between invasive populations and to infer colonization routes within North America.

Though we find limited population structure across space or time in introduced North American populations, we identified 2 selective sweeps in regions containing genes in the cytochrome P450 pathway, implicating pesticide resistance as a potential cause of recent sweeps in the invaded range. These haplotypes were not found in African samples, suggesting these alleles may have evolved in the past ∼25 years since *Z. indianus* invaded the Western Hemisphere, though we can’t rule out their origin in another undescribed population. Additional work will be required to characterize insecticide resistance in *Z. indianus* and compare resistance across alleles to test the hypothesis that these alleles are involved in a recent sweep. We also find a region on the X chromosome that shows potential evidence of a selective sweep in temperate regions, potentially due to copy number variation, but the signal could be an artifact of genome assembly issues and sequencing near repetitive elements. Further investigation will be required to resolve the signals in this region. Studying how genetic variation in this region of the genome influences survival in temperate environments will be an important direction of future research. We additionally find that the X chromosome has an unusually complex evolutionary history in *Z. indianus*. It may have several segregating inversions and CNVs, has strong signatures of selection, and shows regions of high divergence both between African and North American populations and within North America. Specifically, long-read sequencing strategies will be important to understand likely inversions both on the X and throughout the *Z. indianus* genome that are common in the invaded range. Large inversions can link together adaptive alleles and are often important drivers of evolution in rapidly changing environments (Thompson and Jiggins 2014), so these regions will be important to track over larger spatial and temporal scales in future studies.

These results underscore the complexity of genetic dynamics during invasions and the need for further studies to explore the adaptive potential, pesticide resistance, and ecological impacts of *Z. indianus* in its invasive range. One limitation of our study is the sample size for each year and location: our ability to estimate allele frequencies or detect subtle changes in allele frequencies across time or space is limited. Sampling strategies that incorporate more individuals, such as pooled sequencing (Bergland et al. 2014; Kapun et al. 2021; Machado et al. 2021; Nunez et al. 2024), will be required to detect these more subtle changes, if they occur, and to understand how they may contribute to rapid adaptation to new environments. *Z. indianus* provides a unique system in which we can study independent invasion events across multiple years and locations, and continuing studies using the genomic resources presented here may offer insights towards the mechanisms and predictability of rapid evolution of invasive species.

## Supplementary Material

jkaf178_Supplementary_Data

## Data Availability

New individual sequencing data have been deposited in the SRA under project number # PRJNA991922 and SRA accession # SRP447595. RNA sequencing from larval and pupal samples, and larval Hi-C data used for scaffolding are deposited under the same project number. The genome sequence has been deposited at DDBJ/ENA/GenBank under the accession JAUIZU000000000. The metadata for all sequencing samples (including date and location of collection); the annotation information for transcripts, proteins and repeats; and VCFs of SNPs and structural variants have been deposited to Dryad: https://doi.org/10.5061/dryad.q2bvq83v3. All code to reproduce analyses has been deposited to Zenodo and is available at https://doi.org/10.5281/zenodo.13799575. All code for analysis is also available at: https://github.com/ericksonp/Zindianus_individual_sequencing. Supplemental material available at G3 online.
